# 

**Published:** 1862-09

**Authors:** 


### CHICAGO MEDICAL JOURNAL.

**Terms, $2.00 per Annum.**

### CONTENTS OF THE SEPTEMBER NUMBER.

PAGE.

SELECTED.

On the Exhibition of Food in Typhoid Fever........ 494

Cholera Infantum. By W. Owen Brown, M. D., of Providence. Read at the Quarterly Meeting of the Rhode Island Medical Society, Oct. 2d, 1861........ 498

Individual Remedy in Epilepsy........ 506

On Ovariotomy; the mode of its Performance, and the Results Obtained at the London Surgical Home. By J. B. Brown, Esq., F. R. C. S., Senior Surgeon to the London Surgical Home........ 509

Conservative Medicine. By Austin Flint, M. D., Prof. of the Principles and Practice of Medicine in the Bellevue Hospital Medical College, N. Y., and in the Long Island Hospital........ 511

ORIGINAL COMMUNICATIONS.

Case of Ovariotomy—Successful. Reported by A. Fisher, M. D., of Chicago........ 525

Army Correspondence........ 530

EDITORIAL AND MISCELLANEOUS........ 533

